# Supplementary figures and images for: Tailored modulation of S100A1 and RASSF8 expression by butanediamide augments healing of rotator cuff tears
Source: PeerJ. 2023 Aug 14;11:e15791. doi: 10.7717/peerj.15791 (PMC10434103; doi:10.7717/peerj.15791)

A

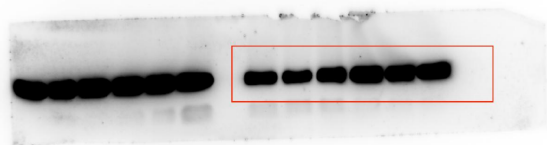

GAPDH

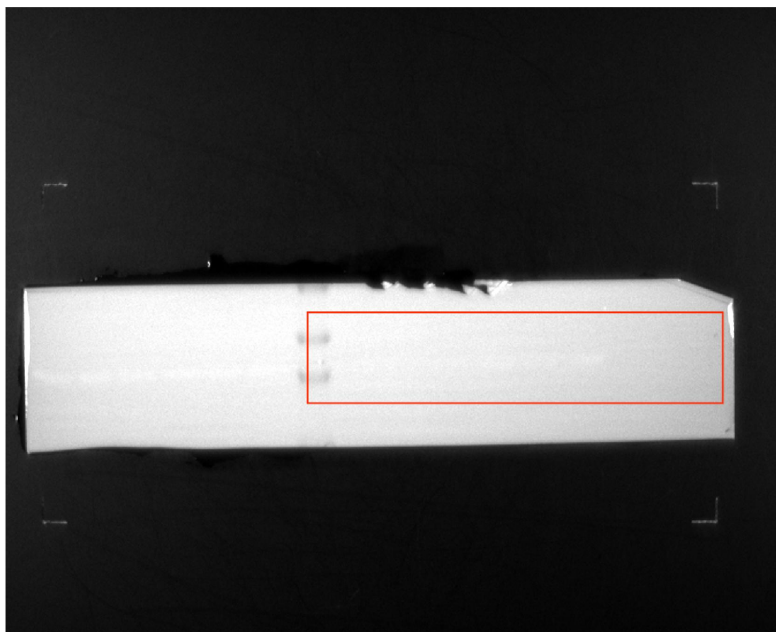

B

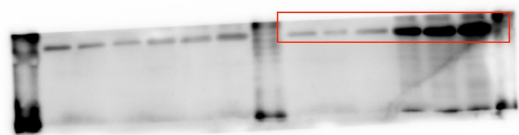

S100A1

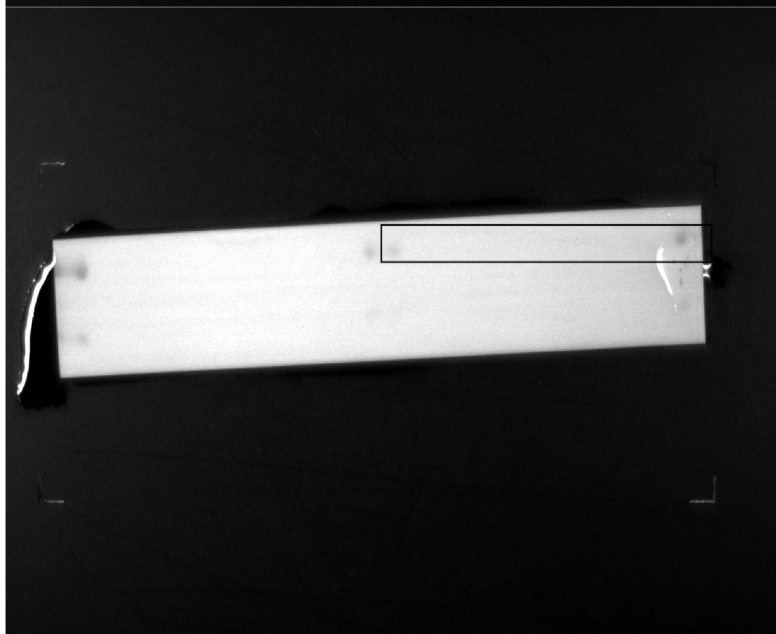

Supplement: Supplemental Information 2 [file peerj-11-15791-s002.pdf]
