# Supplementary material for: Tailored modulation of S100A1 and RASSF8 expression by butanediamide augments healing of rotator cuff tears
Source: PeerJ. 2023 Aug 14;11:e15791. doi: 10.7717/peerj.15791 (PMC10434103; doi:10.7717/peerj.15791)
Supplement: Table S1 [file peerj-11-15791-s004.docx]

**Supplementary Table 1. The top 20 compounds targered to S100A1 according to LibDockScore.**

| **Number** | **Compounds** | **Libdock score** | **Number** | **Compounds** | **Libdock score** |
| --- | --- | --- | --- | --- | --- |
| **1** | ZINC000049841054 | 155.257 | **11** | ZINC000150338699 | 139.975 |
| **2** | ZINC000085537014 | 155.157 | **12** | ZINC000003830635 | 139.912 |
| **3** | ZINC000003941496 | 152.984 | **13** | ZINC000028232750 | 138.847 |
| **4** | ZINC000003944422 | 151.549 | **14** | ZINC000014210876 | 137.241 |
| **5** | ZINC000026985532 (Butanediamide) | 144.975 | **15** | ZINC000029416466 | 136.785 |
| **6** | ZINC000049783788 | 143.821 | **16** | ZINC000022448696 | 136.641 |
| **7** | ZINC000253632968 | 143.103 | **17** | ZINC000029571072 | 136.388 |
| **8** | ZINC000026664090 | 142.195 | **18** | ZINC000095564694 | 134.77 |
| **9** | ZINC000038945666 | 142.057 | **19** | ZINC000095551509 | 134.327 |
| **10** | ZINC000003914596 | 141.427 | **20** | ZINC000003824921 | 134.194 |
