# Supplementary material for: Tailored modulation of S100A1 and RASSF8 expression by butanediamide augments healing of rotator cuff tears
Source: PeerJ. 2023 Aug 14;11:e15791. doi: 10.7717/peerj.15791 (PMC10434103; doi:10.7717/peerj.15791)
Supplement: Table S2 [file peerj-11-15791-s005.docx]

**Supplementary Table 2. The top 20 compounds targered to RASSF8 according to LibDockScore.**

| **Number** | **Compounds** | **Libdock score** | **Number** | **Compounds** | **Libdock score** |
| --- | --- | --- | --- | --- | --- |
| **1** | ZINC000028232750 | 146.405 | **11** | ZINC000049841054 | 135.941 |
| **2** | ZINC000003830635 | 145.626 | **12** | ZINC000009212428 | 135.871 |
| **3** | ZINC000095564694 | 139.8 | **13** | ZINC000000538065 | 134.667 |
| **4** | ZINC000085537014 | 139.564 | **14** | ZINC000150338698 | 134.333 |
| **5** | ZINC000036701290 | 137.788 | **15** | ZINC000022448696 | 133.511 |
| **6** | ZINC000072267023 | 137.324 | **16** | ZINC000001530579 | 133.147 |
| **7** | ZINC000014768621 | 137.067 | **17** | ZINC000029571072 | 132.21 |
| **8** | ZINC000011616925 | 136.504 | **18** | ZINC000038945666 | 131.596 |
| **9** | ZINC000009212427 | 136.483 | **19** | ZINC000100016058 | 131.541 |
| **10** | ZINC000049783788 | 136.06 | **20** | ZINC000100022637 | 131.541 |
