# Supplementary material for: Tailored modulation of S100A1 and RASSF8 expression by butanediamide augments healing of rotator cuff tears
Source: PeerJ. 2023 Aug 14;11:e15791. doi: 10.7717/peerj.15791 (PMC10434103; doi:10.7717/peerj.15791)
Supplement: Table S3 [file peerj-11-15791-s006.docx]

**Supplementary Table 3. Molecular docking results of the first six compounds targeting the core protein S100A1 binding.**

| **Protein** | **Compound** | **Structure** | **Vina(kcal·mol^-1^)** | **RMSD** | **DS(LibDockScore)** | **Hydrogen bond interaction** | **Hydrophobic interaction** |
| --- | --- | --- | --- | --- | --- | --- | --- |
| **S100A1**  **(5K89)** | **ZINC000049841054** | 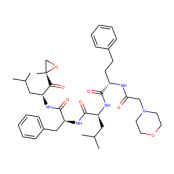 | -5.9 | 1.621 | **155.257** | CYS:86,THR:83 | LEU:12,PHE:45,LEU:37,LEU:38,LEU:42,ALA:8 |
| **S100A1**  **(5K89)** | **ZINC000085537014** | 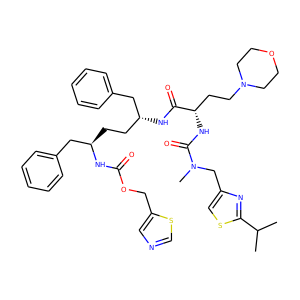 | -5.8 | 1.758 | **155.157** | PHE:45,GLN:39,SER:43 | ALA:85,PHE:45,LEU:82,VAL:79,LEU:42,ALA:8,LEU:37,CYS:86 |
| **S100A1**  **(5K89)** | **ZINC000003941496** | 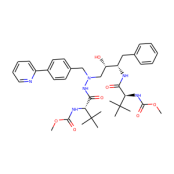 | -6.3 | 1.228 | **152.984** | SER:43,GLN:39,THR:40,THR:83 | LEU:46,CYS:86,ALA:85,LEU:82,LEU:42,TYR:75,LEU:78,LEU:37,VAL:79 |
| **S100A1**  **(5K89)** | **ZINC000003944422** | 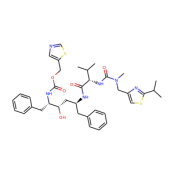 | -5.6 | 1.918 | **151.549** | ASP:47 | LEU:46,CYS:86,ALA:85,LEU:82,VAL:79 |
| **S100A1**  **(5K89)** | **ZINC000026985532 (Butanediamide)** | 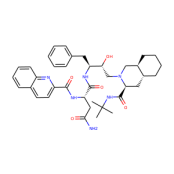 | -7.9 | 2.219 | **144.975** | THR:83 | VA:79,LEU:37,LEU:42 |
| **S100A1**  **(5K89)** | **ZINC000049783788** | 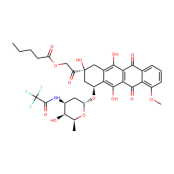 | -6.2 | 2.777 | **143.821** | CYS:86 | LEU:12,VAL:15,VAL:79,LEU:82,PHE:90 |
