# Supplementary material for: Tailored modulation of S100A1 and RASSF8 expression by butanediamide augments healing of rotator cuff tears
Source: PeerJ. 2023 Aug 14;11:e15791. doi: 10.7717/peerj.15791 (PMC10434103; doi:10.7717/peerj.15791)
Supplement: Table S4 [file peerj-11-15791-s007.docx]

**Supplementary Table 4. Molecular docking results of the first six compounds targeting the core protein RASSF8 binding.**

| **Domain** | **Compound** | **Structure** | **Vina(kcal·mol^-1^)** | **RMSD** | **DS(LibDockScore)** | **Hydrogen bond interaction** | **Hydrophobic interaction** |
| --- | --- | --- | --- | --- | --- | --- | --- |
| **RASSF8**  **(2CS4)** | **ZINC000028232750** | 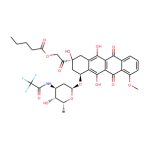 | -5.8 | 2.398 | 146.405 | ARG:86,ARG:85,LEU:84 | TYR:47,ASP:15,TRP:13 |
| **RASSF8**  **(2CS4)** | **ZINC000003830635** | 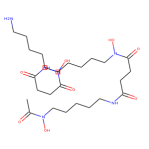 | -3.9 | 2.751 | 145.626 | GLY:16,ASP:15,LEU:84,TRP:13,TYR:47,ARG:86,SER:90 | - |
| **RASSF8**  **(2CS4)** | **ZINC000095564694** | 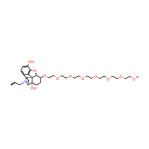 | -4.6 | 2.216 | 139.8 | LYS:52,TRP:13,GLY:16,ARG:85,LEU:84,ASP:15,ILE:20,LEU:37,GLU:57 | - |
| **RASSF8**  **(2CS4)** | **ZINC000085537014** | 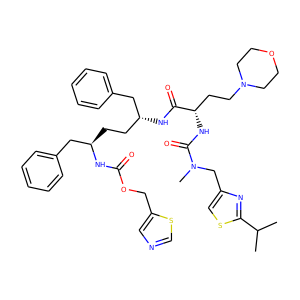 | -5.4 | 2.353 | 139.564 | ARG:85,ASP:15 | ARG:86ILE:83,TRP:13,VAL:14,LEU:84 |
| **RASSF8**  **(2CS4)** | **ZINC000036701290** | 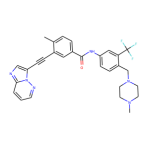 | -6.9 | 1.066 | 137.788 | TRP:13,ARG:43,ARG:86,ARG:85 | LYS:52,ILE:83,LEU:84,VAL:14,ASP:15,TYR:47,ILE:41 |
| **RASSF8**  **(2CS4)** | **ZINC000072267023** | 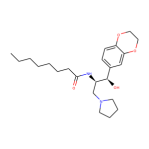 | -4.5 | 1.386 | 137.324 | THR:87,TYR:47 | ARG:46,ILE:35,ALA:38,VAL:34,PRO:62,CYS:30,LEU:49 |
